# Maternal Hyperglycemia Directly and Rapidly Induces Cardiac Septal Overgrowth in Fetal Rats

**DOI:** 10.1155/2015/479565

**Published:** 2015-05-07

**Authors:** Erin E. Gordon, Benjamin E. Reinking, Shanming Hu, Jianrong Yao, Kok L. Kua, Areej K. Younes, Chunlin Wang, Jeffrey L. Segar, Andrew W. Norris

**Affiliations:** ^1^Department of Pediatrics, University of Iowa Carver College of Medicine, Iowa City, IA 52242, USA; ^2^Department of Biochemistry, University of Iowa Carver College of Medicine, Iowa City, IA 52242, USA; ^3^Fraternal Order of Eagles Diabetes Research Center, University of Iowa, Iowa City, IA 52242, USA

## Abstract

Cardiac septal overgrowth complicates 10–40% of births from diabetic mothers, but perplexingly hyperglycemia markers during pregnancy are not reliably predictive. We thus tested whether fetal exposure to hyperglycemia is sufficient to induce fetal cardiac septal overgrowth even in the absence of systemic maternal diabetes. To isolate the effects of hyperglycemia, we infused glucose into the blood supply of the left but not right uterine horn in nondiabetic pregnant rats starting on gestational day 19. After 24 h infusion, right-sided fetuses and dams remained euglycemic while left-sided fetuses were moderately hyperglycemic. Echocardiograms in utero demonstrated a thickened cardiac septum among left-sided (glucose-exposed, 0.592 ± 0.016 mm) compared to right-sided (control, 0.482 ± 0.016 mm) fetuses. Myocardial proliferation was increased 1.5 ± 0.2-fold among left-sided compared to right-sided fetuses. Transcriptional markers of glucose-derived anabolism were not different between sides. However, left-sided fetuses exhibited higher serum insulin and greater JNK phosphorylation compared to controls. These results show that hyperglycemic exposure is sufficient to rapidly induce septal overgrowth even in the absence of the myriad other factors of maternal diabetes. This suggests that even transient spikes in glucose may incite cardiac overgrowth, perhaps explaining the poor clinical correlation of septal hypertrophy with chronic hyperglycemia.

## 1. Introduction

Cardiac septal overgrowth affects 10–40% of neonates born to pregnancies complicated by maternal diabetes [1–6]. The functional impact of neonatal cardiac septal hypertrophy can range from clinically asymptomatic to potentially fatal congestive heart failure stemming from left ventricular outflow tract obstruction. Neonatal cardiac septal hypertrophy (meaning overgrowth) is rare among infants from nondiabetic pregnancy [5, 7], and diabetes during pregnancy imparts an 18-fold relative risk compared to nondiabetic pregnancy [6].

It is unclear what component of the intrauterine milieu perturbed by maternal diabetes is responsible for fetal cardiac septal overgrowth. Because of the close link to maternal diabetes, hyperglycemia is a leading causative candidate. However, the relationship of maternal glycemia to neonatal cardiac septal hypertrophy remains uncertain. Some studies report that elevated maternal glycemia is associated with cardiac septal hypertrophy [3, 8], whereas other studies report no relation [2, 4, 5, 9, 10]. Furthermore, newborns from pregnancies where glucose levels have been tightly controlled are still at risk of septal hypertrophy [11–13]. More surprisingly, newborns of tightly controlled diabetic mothers still have a high incidence of cardiac septal hypertrophy despite having abnormally low glycosylated fetal hemoglobin (HbF_1c_) indicating lower than normal antecedent fetal glucose levels [14]. Because of the uncertain relation between hyperglycemia and neonatal cardiac septal hypertrophy, it has been postulated that other derangements of diabetic pregnancy, such as other elevated maternal fuels, altered placental nutrient transport, or hormonal disturbances, may be responsible for the septal overgrowth [2, 6, 15].

To better understand the interaction between glucose and the fetal myocardium, our laboratory has utilized a recently established rat model [16, 17] that delivers localized hyperglycemia to selected fetus in nondiabetic dams. Using this model of maternal diabetes, we examined the effects of hyperglycemia on the fetal myocardium during late gestation. Importantly, this model isolates the effects of hyperglycemia only, avoiding the myriad effects of maternal diabetes since the dams and control fetuses remain euglycemic throughout [16, 17]. We used this model to test the hypothesis that hyperglycemia is sufficient to induce fetal cardiac septal hypertrophy, finding that even transient hyperglycemic exposure induces septal overgrowth. Because this result directly implicates maternal hyperglycemia in the etiology of septal overgrowth, we examine signaling pathways and gene expression patterns that might contribute to glucose-related overgrowth, finding potential roles for fetal hyperinsulinemia and for JNK activation.

## 2. Materials and Methods

### 2.1. Animals

All animal procedures were performed within the regulations of the Animal Welfare Act and the National Institutes of Health Guide for the Care and Use of Laboratory Animals and were approved by the Institutional Animal Care and Use Committee of the University of Iowa. Timed-pregnant Sprague-Dawley nondiabetic female rats (Hsd: Sprague Dawley SD; Harlan Laboratories, Inc., Indianapolis, IN) were maintained on standard laboratory chow and water ad libitum. Gestational day 0 was defined as the day of initial vaginal plug detection and the normal gestational length for this strain is 21 ± 1 days. On the 19th day of gestation, an infusion catheter was placed as described [16] so as to expose only the fetuses of the left, but not right, uterine horn via the left uterine artery. Glucose (20 g/dL), containing 2 U/mL heparin, was then infused continuously at 4 mg/min via this catheter at a fluid infusion rate of 20 microliters/min. After 24 hours of infusion (i.e., on gestational day 20), fetuses were accessed for echocardiography or blood sampling while continuing the glucose infusion during these procedures. Fetuses were accessed by uterine exteriorization under warm, moist conditions via a midline abdominal incision under surgical anesthesia. Fetuses were studied in a fashion alternating between the left and right uterine horns beginning with those closest to the cervix. Fetal blood sampling was performed via incision of the right subclavian artery with collection into heparinized glass capillary tubes. Immediately following study, anesthetized fetuses were delivered by cesarean section and euthanized. A portion of the fetal hearts was rapidly excised to be frozen in liquid nitrogen and stored at −80°C or placed in 1% formalin.

### 2.2. Echocardiography

Echocardiograms were performed using the Vevo 2100 High Resolution Imaging System and software (VisualSonics Inc., Toronto, Canada). The transducer was placed directly on the uterine wall at the level of each fetus, and the fetal heart was visualized via short axis view of the right and left ventricles. Diastolic thickness of the interventricular septum was measured at the level of the left ventricular papillary muscles. Each ultrasound thickness reported is the mean of 3 consecutive measurements. The measurements were made in accordance with the American Society for Echocardiography Guidelines by a single operator who was blinded as to the nature of the infusate.

### 2.3. Assays

Blood glucose was determined using a One-Touch (LifeScan, Milpitas, CA) meter. Serum was stored at −80°C and later assessed for insulin content by ELISA (#10-1250-01, Mercodia, Uppsala, Sweden).

### 2.4. Growth-Related Kinase Signaling

Several intracellular kinases are proposed to be signaling mediators in the pathogenesis of cardiac overgrowth. Of these, AKT and the MAP-kinases JNK, ERK, and p38 are leading candidates [18–21]. These were assessed by Western blotting of fetal heart homogenized using a MicroGrinder (RPI, Mount Prospect, IL) in 100 *μ*L cell lysis reagent (Sigma: #C2978, St Louis, MO), 1 *μ*L protease inhibitor cocktail (Sigma: #P8340), and 1 *μ*L phosphatase inhibitor cocktail set II (Calbiochem/EMD Biosciences: #524625, San Diego, CA). Antibody immunoassays were performed for total and phosphorylated JNK, total and phosphorylated AKT (Cell Signaling Technology, Danvers, MA), and total and phosphorylated ERK. Band densities were quantified by V750 pro Scanner (Epson, Long Beach, Ca) and IMAGEJ. For Western blotting, in addition to 24-hour infused dams, a separate cohort of dams was studied, which underwent glucose infusion using an identical approach except that the infusion duration was 48 instead of 24 hours, being initiated on gestational day 18 and completed on gestational day 20.

### 2.5. Cardiac Immunofluorescence

Cardiac tissues were fixed in 10% neutral buffered formaldehyde for 24 hours. Dehydrated sections were cut at 8 *μ*m thickness, placed on positively charged microscope slides, dried, deparaffinized with xylene, and rehydrated with graded ethanol. For antigen retrieval, slides were washed twice with PBS, boiled for 20 minutes in 0.1 M Sodium citrate buffer (pH 6.0), and then permeabilized with 0.2% Triton X-100. Bovine serum albumin, 2%, in PBS was used as a blocking agent. Slides were incubated overnight with 100–120 *μ*L rabbit anti-Ki67 antibody (1 : 50) at 4°C. After washing in PBS, Alexa Fluor 488-conjugated secondary antibody (1 : 200) was added and nuclei were stained with To-PRO-3 (1 : 2000). Slides were examined at 40x magnification with a Zeiss 510 confocal microscope, 3 fields per slide. Ratios comparing nuclei with active proliferation (Ki67 stained) to all nuclei (TO-PRO-3 stained) per field were obtained via manual counting. These histopathologic quantifications were performed by one individual who was blinded as to the sample identities.

### 2.6. Measurement of Warburg Effect-Related Gene Expression

A prominent mechanism by which glucose can generate cellular growth is the Warburg effect [22–26]. The Warburg effect increases the flux of glucose carbons into anabolic pathways, thus supporting excess cellular growth. This effect is induced by changes in gene expression that favor specific aspects of anaerobic glucose metabolism. Maternal diabetes favors Warburg conditions by both increasing glucose levels and further reducing fetal oxygen tension [27, 28]. We thus tested the expression of 8 genes known to be induced in the Warburg effect (Table 1). To accomplish this, fetal rat hearts were snap-frozen in liquid nitrogen and homogenized in 1 mL TRIzol (Invitrogen, Carlsbad, CA) for 2 minutes at 50 Hz on a LT TissueLyser using one 7 mm SS bead (Qiagen, Valencia, CA). RNA was isolated via chloroform extraction and isopropanol precipitation according to the TRIzol protocol. RNA, 1 *μ*g, was served to synthesize cDNA using a high-capacity, random-primed reverse transcription kit (product #4368814, Applied Biosystems, Foster City, CA) according to the manufacturer's instructions. RT-PCR primers were designed such that at least one of each primer pair spanned exon-exon boundaries using Primer3plus [29], avoiding amplicon's secondary structures using mFold [30] and avoiding dimer-primer and hairpin structures using Beacon Designer Free Edition (Premier Biosoft, Palo Alto, CA). Primers were synthesized by IDT DNA technology (Coralville, IA, USA). Power SYBR green PCR Master Mix (#4367659, Life Technology) was used for qPCR reactions on 156 nM final primer concentrations and 7 ng cDNA. The reaction was performed on a Bio-Rad (Hercules, CA) CFX96 thermal cycler with the following temperature program: 95°C, 10 min, 40 cycles (60°C, 15 s → 72°C, 1 min) then melting curve program (95°C, 10 s, then 60°C to 95°C, 0.5°C increment). The efficiencies of all primers were verified to be between 95 and 105%, using cDNA dilutions spanning 5 magnitudes. The relative amount of RNA was calculated in reference to *β*-actin using the 2^−ΔΔCT^ method.

### 2.7. Statistical Analysis

The significance of the differences between nonpaired groups was assessed by Student's unpaired *t*-test with significance defined as a *P* < 0.05. Paired, balanced comparisons were performed for Western blot results on a per-dam basis, comparing the mean values between uterine horns by paired *t*-test. The statistical significance of differences involving unbalanced paired comparisons were assessed by resampling as described [17].

## 3. Results

### 3.1. Induction of Fetal Hyperglycemia

**Figure 1:**
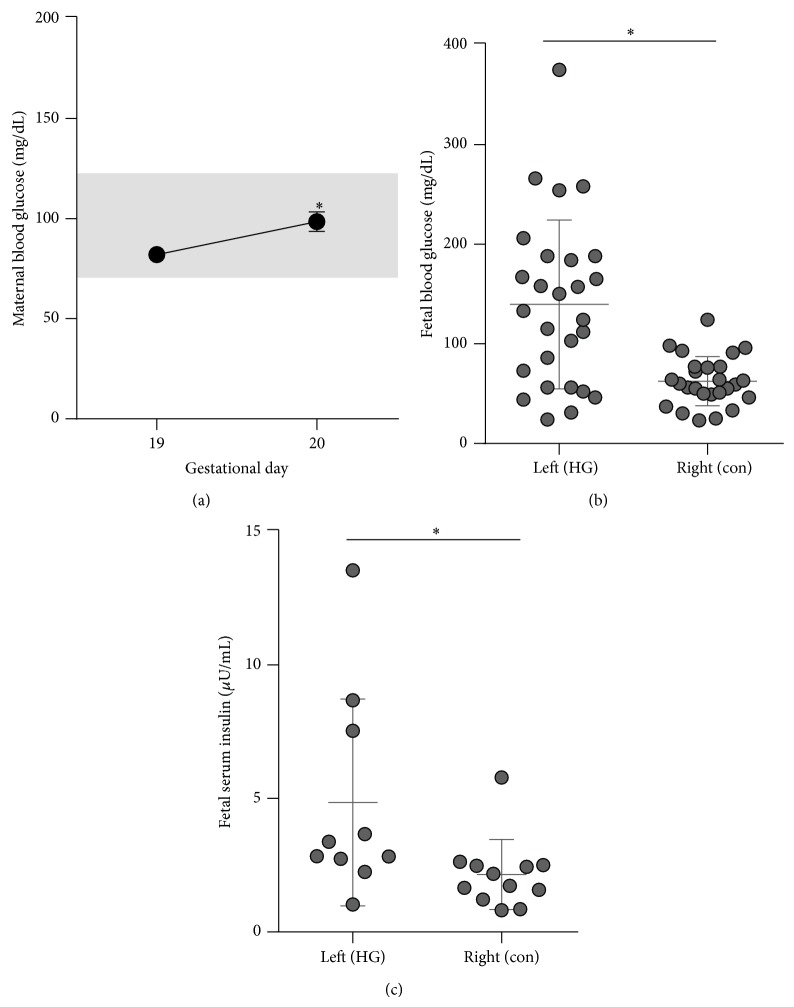
Maternal and fetal glucose and insulin levels. Glucose was infused into the left uterine artery from gestational day 19 until day 20. (a) Maternal blood glucose was measured in the nonfasted state just prior to infusion on gestational day 19 and just before the conclusion of the infusion on gestational day 20. The euglycemic range (70–130) is highlighted on the figure in grey. ^∗^ *P* < 0.001 by paired *t*-test, *N* = 10 dams. (b) Fetal blood glucose was measured just before the conclusion of the infusion for right-sided (i.e., control, “con”) and left-sided (i.e., hyperglycemia infused, “HG”) fetuses. ^∗^ *P* = 0.05 by paired *t*-test, *N* = 5 dams, 26-27 fetuses. (c) Fetal serum insulin just before the conclusion of the infusion. ^∗^ *P* < 0.05 by *t*-test, *N* = 4 dams, 10–12 fetuses.

Pregnant dams were infused with 4 mg/min dextrose such that hyperglycemia exposure occurred in the left but not right uterine horn blood supply. The infusion was initiated on gestational day 19 and continued through the conclusion of the study on gestational day 20. Maternal blood glucose remained euglycemic during the infusion with a small but significant increase in maternal glycemia by the conclusion of the infusion as compared to preinfusion (Figure 1(a)). Fetuses were examined at 24 hours of infusion. At this time, blood glucose was lower in the right-sided fetuses (57 ± 8 mg/dL) than in maternal blood (102 ± 4). These right-sided fetal blood glucose levels would be considered hypoglycemic by postnatal standards but are considered euglycemic for late gestational fetal rats which typically have mean blood glucose levels near 50 mg/dL [31–34]. By contrast, left-sided fetuses had 128 ± 58% higher glucoses (131 ± 33 mg/dL) than right-sided fetuses (Figure 1(b)). Fetal insulin was increased 125 ± 58% among left as compared to right-sided fetuses (Figure 1(c)). Maternal blood glucose, per dam, is shown versus fetal blood glucose and weight in Tables 2 and 3, respectively.

### 3.2. Cardiac Overgrowth

**Figure 2:**
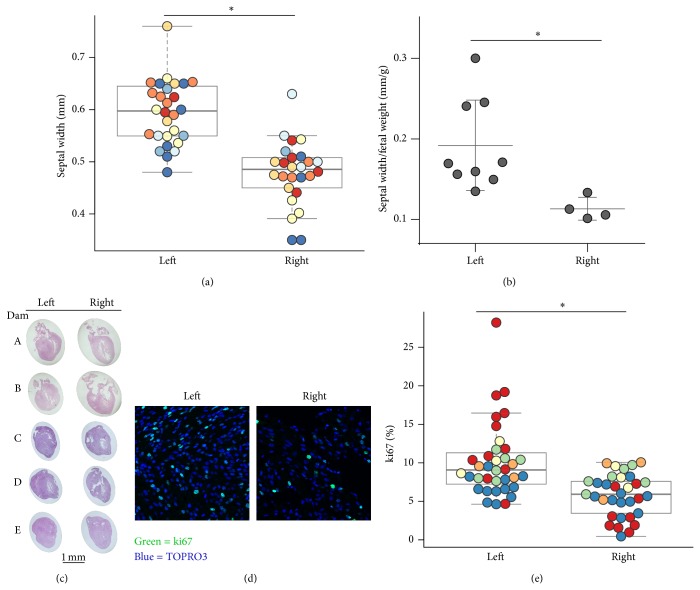
Cardiac septal width and myocardial proliferation. (a) Fetal cardiac septal width, as measured by echocardiograph, among left-sided (i.e., hyperglycemia infused) and right-sided (control) fetuses. Differing colors indicate different dams. ^∗^ *P* < 0.0005 by resampling, *N* = 7 dams, *N* = 26, 28 fetuses per side. (b) The ratio of cardiac septal width to fetal weight. ^∗^ *P* < 0.05 by *t*-test, *N* = 4, 9 fetuses per side, 2 dams. (c) Hematoxylin and eosin stained fetal hearts from the left (hyperglycemia) and right (control) uterine horns. (d) Myocardial proliferation as evidenced by immunofluorescence detection of ki67 (green) and DAPI (blue). Representative images from the interventricular septal region are shown. (e) The percentage of ki67 positivity among nuclei in the fetal myocardium. ^∗^ *P* < 0.0005 by resampling, *N* = 5 dams, >500 nuclei from 3 myocardial regions (left, right, septal ventriculum) analyzed per fetus. *N* = 33, 36 fetuses per side.

The primary outcome of interest was whether the 24 hours of hyperglycemia among left-sided fetuses was sufficient to alter the cardiac septal width as compared to the right-sided (euglycemic) fetuses. Thus the diastolic interventricular cardiac septum was measured by echocardiography in exteriorized fetuses on gestational day 20 after 24 hours 4 mg/min dextrose infusion. The septal thickness was 22.8 ± 3.2% greater among left-sided (hyperglycemia exposed, 0.592 ± 0.016 mm) fetuses as compared to that of right-sided (control, 0.482 ± 0.016 mm) fetuses (Figure 2(a)). Likewise, septal thickness normalized to fetal weight was greater among left-sided fetuses (0.19 ± 0.02 mm/g) compared to right-sided fetuses (0.11 ± 0.01 mm/g) (Figure 2(b)), indicating the septal overgrowth exceeded general body growth. Microscopic sections from left-sided fetuses exhibited relative ventricular and septal wall thickness such that the ventricular cavity space was reduced and often not even visually evident, as compared to that of right-sided fetuses (Figure 2(c)). Total cardiac weight did not differ between left- and right-sided fetuses (*P* = ns, *N* = 4 dams, 9–33 fetuses). We assessed whether the increased myocardial septal thickness was associated with altered myocardial proliferation by determining which nuclei expressed Ki-67, a nuclear protein present during active portions of the cell cycle but absent from cells resting in the resting (*G*_0_) phase. Interestingly, the percent of nuclei positive for Ki67 was 51 ± 18% higher in the septal myocardium of left-sided compared to right-sided fetuses (Figures 2(d) and 2(e)).

### 3.3. Impact of Fetal Hyperglycemia on Glucose Anabolism-Related Gene Expression

The above results show that hyperglycemic exposure is sufficient to induce fetal cardiac septal overgrowth, suggesting the possibility that hyperglycemia might directly induce fetal myocardial growth. Glucose can generate cellular growth via the Warburg effect which increases the anaerobic flux of glucose carbons into anabolic pathways. Since maternal diabetes promotes fetal Warburg conditions by both increasing glucose levels and reducing fetal oxygen tension, we wondered whether a Warburg-like shift in gene expression might contribute to the cardiac overgrowth observed in our model. We thus tested the expression of 8 genes known to be induced in the Warburg effect (Table 1). The expression of all these genes was unchanged or even reduced in hyperglycemia exposed hearts (Table 4). These results suggest that a shift to Warburg-like metabolism is not responsible for the fetal cardiac overgrowth induced by hyperglycemic exposure.

### 3.4. Effect of Hyperglycemia on the Mitogenic Signaling Factors

**Figure 3:**
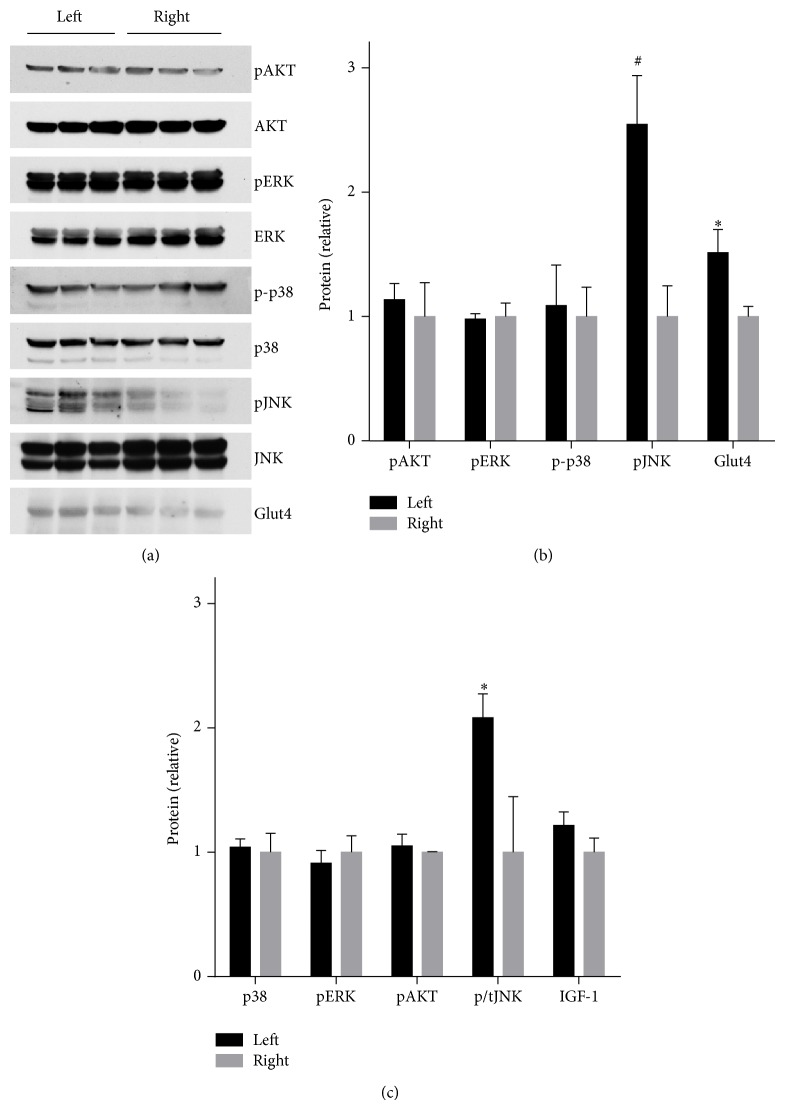
Myocardial protein levels in the heart from left (hyperglycemia exposed) and right (control) fetuses. (a) Western blot of phosphokinase and total kinase levels (AKT, ERK, p38, and JNK) and glucose transporter 4 (Glut4). (b) Quantitation of Western blot results. *N* = 4 dams, 6 fetuses per side. ^∗^ *P* < 0.01, ^#^ *P* < 0.005. (c) Quantitation of Western blot results. *N* = 1, 2,3, 2,3 dams and 4,8, 12,3, 12 fetuses/side for phospho-p38, phospho-ERK, insulin stimulated phospho-AKT, phospho-JNK normalized to total, and IGF-1, respectively. ^∗^ *P* < 0.01. (a-b) Dams infused with left uterine artery hyperglycemia on gestational days 19-20. (c) Dams infused with left uterine artery hyperglycemia on gestational days 18–20.

We investigated the expression levels and activation of intracellular kinases implicated in cardiac overgrowth in the hyperglycemia exposed fetal heart. Hyperglycemic exposure had no effect on the phosphorylation of AKT, ERK, or P38. By contrast, there was a 2.5 ± 0.4 fold induction of phosphorylated JNK levels as a result of hyperglycemic exposure (Figures 3(a) and 3(b)). As confirmation of this result, we studied the effect of longer duration of hyperglycemic exposure in a separate cohort of dams infused for 48 hours on gestational days 18–20. JNK signaling activation was maintained at least through 48 hours of hyperglycemic exposure (Figure 3(c)). We also investigated the expression of IGF-1, a paracrine factor highly implicated in cardiac overgrowth [35], and Glut4, a major glucose transporter in the heart and modulator of cardiac growth [36]. Interestingly, Glut4 expression was mildly increased by hyperglycemia exposure, whereas IGF-1 levels were unchanged (Figure 3).

## 4. Discussion

Although the association of maternal diabetes and newborn cardiac overgrowth has been recognized since the 1940s [37], the causative relationship between maternal hyperglycemia and cardiac overgrowth has been unclear. This uncertainty is highlighted by numerous epidemiologic studies that find no relationship between the degree of maternal hyperglycemia and cardiac overgrowth [2, 4, 5, 9, 10]. Herein, we directly tested the hypothesis that maternal hyperglycemia is sufficient to induce fetal cardiac septal overgrowth by using a recently developed model whereby glucose is infused into the left uterine artery [16]. This approach isolates the effects of hyperglycemia to just the fetuses developing in left uterine horn, with the fetuses in the right horn serving as controls. The cardiac septal width of the hyperglycemia exposed fetuses was significantly greater than the control fetuses, showing that indeed maternal hyperglycemia alone is sufficient to induce cardiac septal overgrowth. This result is consistent with those epidemiologic studies that do find that the degree of maternal hyperglycemia predicts newborn cardiac overgrowth [3, 8].

It is thus an open question as to why multiple studies have not observed a relationship between maternal hyperglycemia and newborn cardiac overgrowth. One possibility that has been forwarded is that other derangements of diabetic pregnancy, such as ketonemia [38] or hyperlipidemia [39], contribute to the fetal septal overgrowth. The studies reported in this paper are aimed at glucose alone and thus do not address these alternative possibilities. It thus remains possible that nonglycemic aspects of diabetic pregnancy may contribute to septal overgrowth, in concert with or independent of hyperglycemia. The rapidity by which hyperglycemia induced fetal cardiac overgrowth was surprising. This informative result however suggests a possible reason for the apparent lack of association between markers of chronic maternal hyperglycemia and fetal septal overgrowth. Namely, it suggests that only transient episodes of hyperglycemia are sufficient to induce septal overgrowth and that chronic hyperglycemia may not be required. Though this finding could have clinical implications in terms of avoiding even transient spikes of hyperglycemia during pregnancy, we consider that application of this implication to the clinical realm is currently premature and requires additional translational studies. For example, it is not clear how brief of a hyperglycemic exposure might be required to induce human fetal cardiac overgrowth. Using our model system it is possible, albeit labor intensive, to define the minimal length of exposure required to induce cardiac overgrowth in the fetal rat. However, there is no clear approach to correlate such a length to the human fetal situation.

The mechanisms underlying diabetes-induced fetal cardiac overgrowth are unclear. One interesting mechanistic possibility is that glucose directly promotes cardiac overgrowth. A well described mechanism by which glucose directly enhances cellular growth is the Warburg effect, which supports the anabolic state of malignant cells. Our results indicate that hyperglycemic exposure does not induce a Warburg-like shift in gene expression in the fetal myocardium. Because the fetal environment is hypoxic, anabolic, and glycolytic at baseline [27, 28], it is possible that the fetal myocardium may already be in a Warburg-like state at baseline and thus susceptible to glucose driven overgrowth. Our experiments cannot exclude this possibility. Another important mechanism in cardiac overgrowth is activation of growth/stress signaling kinases including AKT and MAP-kinases JNK, ERK, and p38 [18–21]. Of these, we found that cardiac JNK is activated by fetal hyperglycemic exposure. JNK has been implicated in the pathogenesis of cardiac hypertrophy during postnatal life [40, 41] including in response to diabetes [19]. On the other hand, JNK has been implicated in contributing to the resolution of cardiac overgrowth [42, 43]. It is thus unclear which of these two roles, detrimental or adaptive, is played by JNK activation in our model. It is possible that studies employing JNK inhibitors could aid in distinguishing between the two opposing possibilities. We found increased levels of Glut4 in hyperglycemia exposed fetal heart, whereas profound deficiency of Glut4 is described to induce cardiac hypertrophy [36]. Little is known about how diabetes affects the expression of Glut4 in fetal tissues, although increased [44] or decreased [45] Glut4 expression has been found in human placenta from diabetic pregnancies. Although IGF-1 levels are increased in fetal serum in diabetic pregnancy [46] and IGF-1 is considered to be a major factor promoting cardiac growth, its expression was not altered in hyperglycemia hearts in our model. There is very little published about the impact of maternal diabetes on the expression of Glut4 and IGF-I in the fetal heart, and thus it is not possible to speculate how maternal diabetes might differ from hyperglycemia alone in this regard. The majority of prior work on cellular signaling during cardiac overgrowth has been conducted in the adult heart or in cultured neonatal cardiac myocytes. It is possible that the signaling events that underlie fetal cardiac overgrowth* in vivo* differ from those in play during postnatal life.

The fetal heart is an insulin-responsive tissue [47, 48] and it has long been hypothesized that elevated fetal insulin induced by maternal diabetes may contribute to fetal cardiac overgrowth [1]. Insulin signaling contributes to both embryonic and postnatal cardiac growth [49]. Furthermore, excess insulin signaling contributes to cardiac hypertrophy and dysfunction [50]. In fact, newborns with primary hyperinsulinism exhibit hypertrophic cardiomyopathy [51]. Fetal insulin levels during exposure to maternal diabetes are often elevated due to the fetal beta-cell response to hyperglycemia. For this reason, it has been difficult to determine whether it is fetal hyperglycemia, fetal hyperinsulinemia, or both that drives cardiac overgrowth in response to maternal diabetes. Our present results likewise do not distinguish between these possibilities since both glucose and insulin are elevated in the left uterine horn fetuses. Thus, it is possible that insulin may be a key factor in the observed cardiac septal overgrowth.

Diabetes-induced fetal cardiac overgrowth has long been described as “hypertrophy” [37], dating prior to the advent of modern techniques that measure cell replication. Myocardial growth during fetal development occurs primarily through cardiac myocyte proliferation, whereas postnatal heart growth occurs through hypertrophy [52]. Our current results indicate that the heart septal overgrowth is accompanied by increased replication, suggesting cardiac hyperplasia. This result is consistent with recent studies also demonstrating fetal cardiac hyperplasia in diabetic pregnant mice [53]. In the present paper we have favored the term “overgrowth” rather than the classic term “hypertrophy” to indicate the uncertainty as to whether the septal enlargement involves just hyperplasia or also hypertrophy. This is an important distinction since the mechanisms that underlie cardiac hyperplasia may be different from those that produce hypertrophy. Our recent findings that cell cycle genes are perturbed in a different model of prenatal cardiac overgrowth speak to this possibility [54].

Our study occurred during late gestation in the fetal rat. At this stage, the heart is structurally fully developed and has been compared to human midgestation based on cardiac morphometry [55] or second-half gestation based on myofibril content [56]. Importantly, the ability of hyperglycemia to promote cardiac overgrowth may be limited to distinct developmental cardiac stages.

Our experimental model system has advantages and disadvantages compared to other* in vivo* models of diabetic pregnancy. One advantage is the ability to isolate the effects of maternal hyperglycemia alone. Another advantage is complete temporal control over the timing of hyperglycemic exposure. Disadvantages to our model include its labor intensive and challenging surgical nature. Additionally, the total hyperglycemic exposure and excess glucose received per fetus cannot be directly measured. A rough estimate can be extrapolated from glucose tracer experiments [16] showing that each left-sided rat fetus receives approximately 2.3% of the infused glucose tracer, which summed over 24 hours of infusion at 4 mg/min amounts to ~130 mg excess glucose per fetus. Although our model system isolates the local maternal effects of hyperglycemia on the fetus, this is not equivalent to producing hyperglycemia alone in the fetus as there will be a number of fetal responses and even local maternal responses to the hyperglycemia. Presumably the hyperglycemia-induced cardiac overgrowth is reversible, just as in human and rodent fetal diabetic cardiac overgrowth, although this was not examined in our model. In human diabetic pregnancy, blood glucose levels often fluctuate considerably, and it is thus possible but not tested that pulsatile infusion of glucose might also result in cardiac overgrowth. Along these lines, an interesting experimental possibility introduced by our model system is to study the acute signaling events that occur in response to the sudden appearance or removal of hyperglycemic exposure. Such studies may help better understand the short and long term effects of fetal hyperglycemic exposure.

In summary, our results demonstrate that maternal hyperglycemia is sufficient to rapidly induce cardiac septal overgrowth. Only transient hyperglycemic exposure is required, possibly explaining why markers of chronic hyperglycemia often do not predict the degree of overgrowth. Increased insulin levels and JNK activation may contribute to this process.

## Figures and Tables

**Table 1:** List of RT-PCR primers.

Gene	NCBI GeneID	Forward	Reverse	Amplicon size (bp)
*Glut1 *	24778	CTTTGGCAGGCGGAACTC	TTGCCCAGTTTGGAGAAACC	84
*Actb *	81822	TTCCTTCCTGGGTATGGAATCC	GGATGTCAACGTCACACTTCATG	77
*Hk2 *	25059	TGGGCTGGACAACCTCAAAG	CCTTGGCAAAGTGAGGATGAAG	74
*Pdk1 *	116551	GCGAGACGGCTTTGTGATTTG	CCTGGTGATTTCGCATTTAGTTC	77
*P53 *	24842	CACTCCAGCTACCCGAAGAC	GCCAGGAACCAGTTTGCATAG	259
*Ldha *	24533	AATGAAGGACTTGGCTGATGAG	GCCATGCTGAAGATCCATCATC	85
*Tigar *	502894	ACGCCTTCTCCAGTGATCTC	CGTACATCCTTTCCCGAAGTC	120
*Gpt2 *	307759	CCCACAGGCCAGGTACAAAG	AAAGCTTCTCTTCCCAGGCAAAG	70
*Pkm2 *	25630	TGACACCTTCCTGGAACACATG	GGGAAGCAGGGCCAATGG	98

Gene abbreviations: *Glut1*, solute carrier family 2 (facilitated glucose transporter) member 1; *Actb*, beta actin; *Hk2*, hexokinase 2; *Pdk1*, pyruvate dehydrogenase kinase isozyme 1; *P53*, tumor protein p53; *Ldha*, lactate dehydrogenase A; *Tigar*, fructose-2,6-bisphosphatase TIGAR; *Gpt2*, glutamic pyruvate transaminase (alanine aminotransferase) 2; *Pkm2*, pyruvate kinase muscle.

**Table 2:** Per dam maternal and fetal blood glucoses.

Maternal blood glucose (mg/dL)		^a^Fetal blood glucose (mg/dL) (mean ± SEM)	
^b^GD19	GD20	Left	Right
98	124	244 ± 132	56
78	97	173 ± 15	46
76	80	28 ± 3	37 ± 5
97	112	126 ± 24	75 ± 4
88	113	168 ± 18	74 ± 12

Each row represents a separate dam. Fetal blood glucose levels without a SEM are from a single fetus. ^a^Measured on gestational day 20. ^b^GD, gestational day.

**Table 3:** Per dam maternal blood glucose and fetal weight.

Maternal blood glucose (mg/dL)		^a^Fetal weight (g) (mean ± SEM)	
^b^GD19	GD20	Left	Right
78	94	3.748 ± 0.082	3.880 ± 0.065
74	103	3.165 ± 0.185	3.650 ± 0.089
75	115	2.890 ± 0.239	3.128 ± 0.146
81	110	2.810 ± 0.070	3.622 ± 0.058
88	113	3.510	3.667 ± 0.081

Each row represents a separate dam. ^a^Measured on gestational day 20. ^b^GD, gestational day.

**Table 4:** Relative expression of genes related to Warburg metabolism.

Gene	Right		Left		*P*
	Ave ± SEM	*N*	Ave ± SEM	*N*	
*Tigar *	1.00 ± 0.06	5	1.07 ± 0.08	6	0.47
*Pkm2 *	1.00 ± 0.10	5	1.03 ± 0.07	6	0.80
*Gpt2 *	1.00 ± 0.05	5	1.07 ± 0.05	6	0.35
*Hk2 *	1.00 ± 0.08	5	0.74 ± 0.11	6	0.10
*Pdk1 *	1.00 ± 0.08	5	0.89 ± 0.08	6	0.39
*P53 *	1.00 ± 0.11	5	1.01 ± 0.07	6	0.93
*Ldha *	1.00 ± 0.07	5	1.06 ± 0.05	6	0.49
*Glut1 *	1.00 ± 0.12	5	1.02 ± 0.09	6	0.92

Expression of genes related to Warburg metabolism. Relative mRNA levels in fetal myocardium were determined by real time PCR. *P* is nonsignificant for all genes tested, *N* = 5-6 fetuses per side, 2 dams. Gene abbreviations are identical to those in Table 1.
